# Modified ilioinguinal approach in combined surgical exposures for displaced acetabular fractures involving two columns

**DOI:** 10.1186/s40064-016-3316-9

**Published:** 2016-09-19

**Authors:** Peng Wang, Xiaodong Zhu, Peng Xu, Yan Zhang, Lubo Wang, Xiangyan Liu, Weidong Mu

**Affiliations:** 1 Department of Traumatic Orthopaedics, Shandong Provincial Hospital Affiliated to Shandong University, 324 Jing Wu Road, Jinan, 250012 Shandong China; 2 Department of Orthopaedics, Weihai Municipal Hospital, Weihai, Shandong China; 3 Department of Orthopaedics, Affiliated Hospital of Binzhou Medical University, Binzhou, Shandong China; 4 Department of Thoracic Surgery, Shandong Provincial Hospital Affiliated to Shandong University, Jinan, Shandong China

**Keywords:** Minimally invasive, Ilioinguinal approach, Kocher–Langenbeck approach, Acetabular fracture

## Abstract

The purpose of this study is to assess the advantages of modified ilioinguinal approach in combined surgical exposures for displaced acetabular fractures involving two columns management. 73 patients with displaced acetabular fractures involving two columns underwent open reduction and internal fixation through combined surgical approaches between 2006 and 2014 in our hospital. The modified ilioinguinal approach combined with Kocher–Langenbeck approach group (group A) included 46 patients. The standard ilioinguinal approach combined with Kocher–Langenbeck approach group (group B) included 27 patients. Outcome was assessed in operative time, blood loss, function outcomes and complications. In group A, the average operative time was 123.2 min, and the average blood loss was 586.2 ml. Anatomic reduction was achieved in 39 patients (84.8 %). The functional recovery was good in 37 patients (80.4 %). Complications related to the approach were observed in 10 patients (21.7 %). In group B, the average operative time was 161.5 min, and the average blood loss was 830 ml. Anatomic reduction was achieved in 24 patients (88.9 %). The functional recovery was good in 22 patients (81.5 %). Complications related to the approach were observed in 9 patients (33.3 %). This study demonstrates that both combined approaches permits good postoperative function results for treatment of acetabular fractures involving two columns. However, the modified ilioinguinal approach combined with Kocher–Langenbeck approach provides less operative time, blood loss and complications.

## Background

Surgical treatment for acetabular fractures is difficult and technically demanding. Most of the acetabular fractures can be treated with a single operative approach (Grubor et al. 2015). However, there are four types complex acetabular fractures involving two columns including transverse fractures, associated transverse and posterior wall fractures, T-shape fractures and both-column fractures, according to the Judet and Letournel classification system (Letournel 1980). Treatment for this complex situation is particularly challenging. The combination of an anterior ilioinguinal and posterior Kocher–Langenbeck approach is usually considered. Routine approaches had serious complications such as increased morbidity due to longer operative time, injuries to the inguinal neurovascular bundle or concomitant lymphatic structures, greater blood loss, infection, abductor weakness, hernias and heterotopic ossification (Matta 1994; Kloen et al. 2002; Helfet and Schmeling 1994; Rommens et al. 1997). To reduce complications related with approach, several modifications approaches are accepted alternatives, especially anterior modifications based on the ilioinguinal approach (Jakob et al. 2006). The purpose of this study is to assess clinical efficiency using the modified ilioinguinal approach combined with Kocher–Langenbeck approach for displaced acetabular fractures involving two columns. We hypothesize that this minimally invasive anterior approach in combined surgical exposures provides: (1) less invasive soft-tissue dissection for less operative time and blood loss, (2) good postoperative functional outcomes, and (3) less postoperative complications.

## Methods

### Patients

**Fig. 1:**
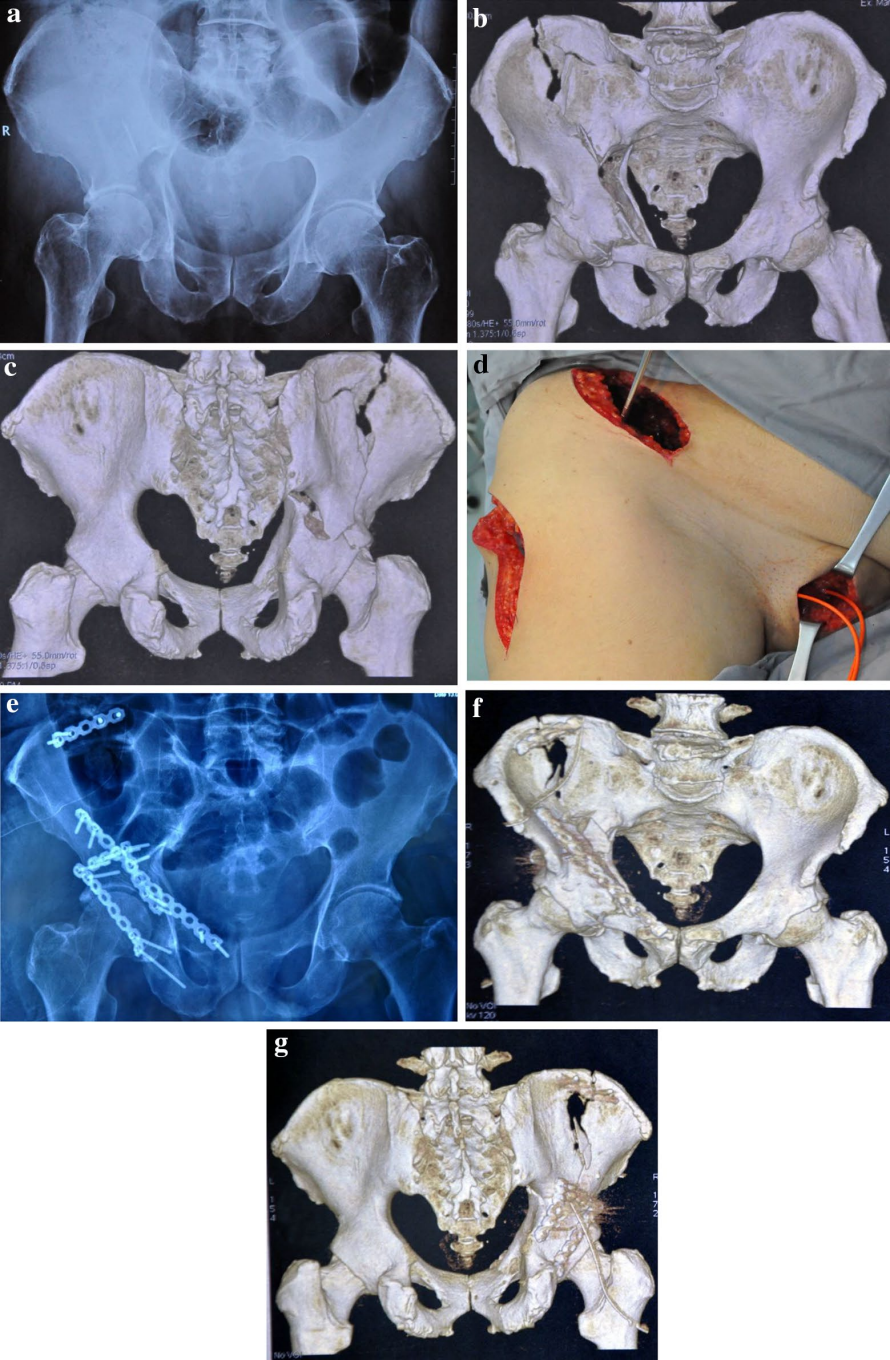
**a** Preoperative diagnostics of an acetabular fracture in a 65-year-old man by falling injury. **b**, **c** 3D CT scan demonstrating both-column acetabular fractures. **d** Intraoperative view of the modified ilioinguinal approach combined with a K–L approach. **e** Postoperative anteroposterior radiograph showing anatomic reduction of the acetabular fractures. **f**, **g** Postoperative 3D CT scan showing anatomic reduction of the acetabular fractures

A total of 73 cases of displaced acetabular fractures involving two columns requiring combined approach operation between 2006 and 2014 in our institution were included. There were 55 males and 18 females. 41 patients were injured by motor vehicle accidents, 26 patients fell from height, and 6 patients were injured by other reasons. According to the Judet and Letournel classification, there were 21 transverse fractures, 11 associated transverse and posterior wall, 15 T-shape fractures and 26 both-column fractures. Pelvic radiographs, computed tomography (CT) scans and three-dimensional reconstruction, were applied in all cases. All patients underwent femoral condyle or tibia tubercle skeletal traction after admission. All the patients were operated on within 3 weeks. 46 patients were operated through modified ilioinguinal approach combined with Kocher–Langenbeck approach (group A) (Fig. 1). The other 27 patients were operated through the standard ilioinguinal approach combined with Kocher–Langenbeck approach (group B). The average age was 46.2 ± 3.12 years in group A, and 44.9 ± 2.36 years in group B. All patients were regularly followed-up. The mean follow-up time was 2.3 (±1.5) years.

### Operative techniques

**Fig. 2:**
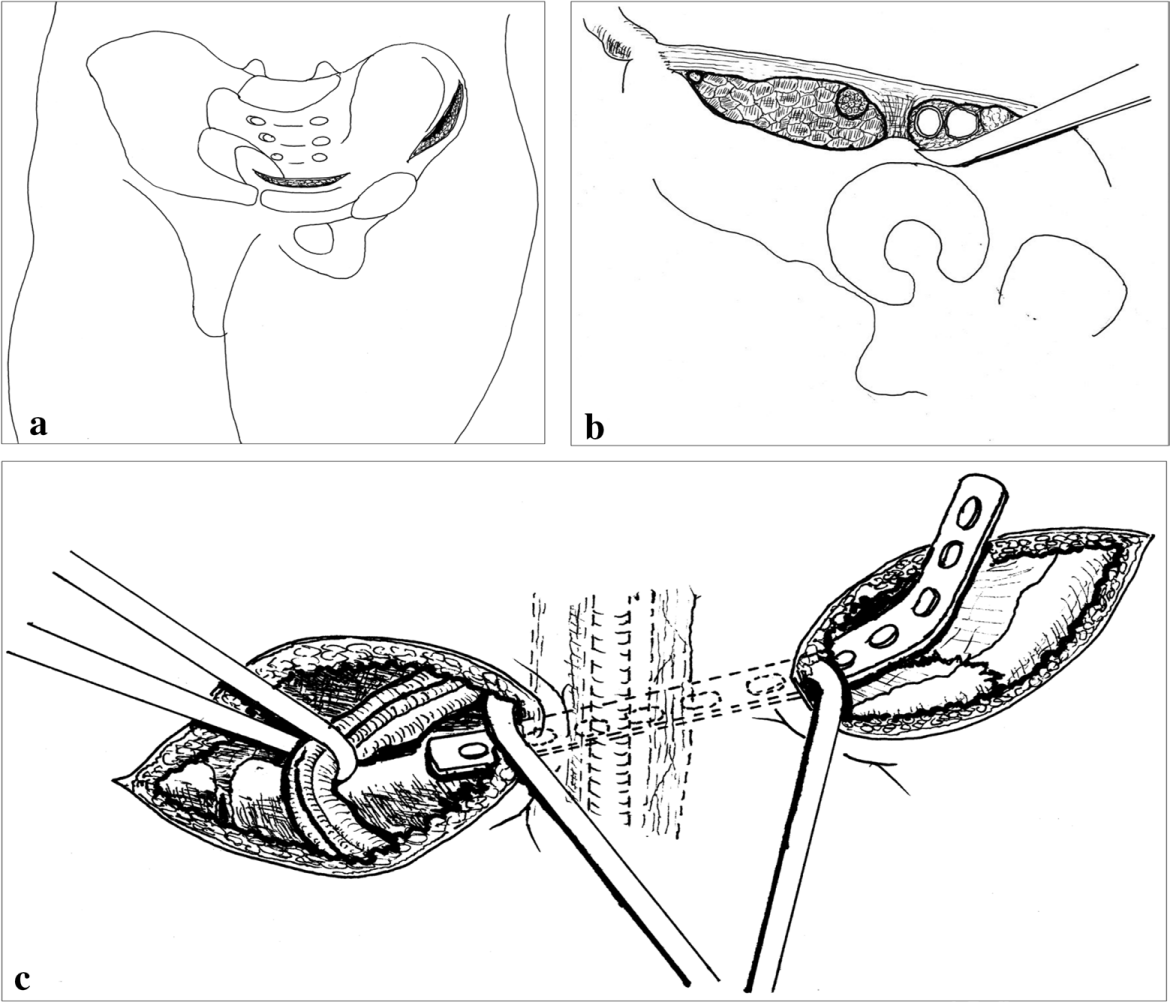
**a** A Pfannenstiel incision was made 1–2 cm above the pubic symphysis and the lateral window used in the ilioinguinal approach but ended just at 1–2 cm lateral to the contour of the femoral artery. **b** Subperiosteal separation of the femoral vascular and the iliopsoas muscles together with the femoral nerve were performed using a blunt periosteal elevator. **c** The spermatic cord or round ligament was protected with a urinary catheter. The pre-bending plate was inserted through the subperiosteal “tunnel” between the two windows

The patient was positioned “float” position on a flat radiolucent operative table for the radiographic projections. The modified ilioinguinal approach combined with the Kocher–Langenbeck approach were used in group A. Standard ilioinguinal approach combined with the Kocher–Langenbeck approach were used in group B. The sequence of exposure depends on the amount of major displacement. The standard ilioinguinal approach or the Kocher–Langenbeck was performed in the routine way. The modified ilioinguinal approach was performed as following. This approach was composed of the lateral and the medial windows (Fig. 2a). Briefly, the lateral incision extended along the anterior two-thirds of the iliac crest as Letournel descripted (Letournel 1993) and ended just at 1–2 cm lateral to the contour of the femoral artery ensuring the internal iliac fossa exposure. The lateral femoral cutaneous nerve was dissected and protected. The abdominal and iliacus muscle from their origin was released from the iliac crest. The iliacus muscle was subperiosteal elevated from the internal iliac fascia. The exposure from the sacroiliac joint to the lateral parts of the pelvic brim was achieved. Then, the medial window through a Pfannenstiel incision was made, 1–2 cm above the symphysis instead of medial portion of ilioinguinal approach. The rectus abdominis and pyramidalis muscles were split longitudinally, extending to the symphysis. The spermatic cord or round ligament were separated and protected. The most part of starting point of the rectus abdominus muscle on the anterior aspect of the pubic bodies was left intact. With the hip and knee joint in nearly 90° flexion, the periosteal elevator was inserted from the lateral window to the medial incision (Fig. 2b). After anatomical fracture reduction and temporarily fixed with K-wires, a preshaped 3.5 mm reconstruction plate was inserted and fixed with screws through the medial and lateral incisions respectively (Fig. 2c).

Postoperative antibiotics were continued for 24 h and drains were removed 24–48 h postoperatively. Patients routinely received deep vein thrombosis prophylaxis for 4 weeks. All patients’ foot-flat weight bearing was not allowed for at least 6–8 weeks depending on the type of the injury.

### Outcome evaluation

Parametric data, such as operative time and blood loss, were expressed as means ± standard deviations and compared using Student’s t test. Nominal variables were expressed as % values and were evaluated using Fisher Exact test. Analysis of the data was performed using SPSS 20.0 (SPSS, Inc., Chicago, IL). A p value of <0.05 was considered statistically significant. Reduction of the articular surface was graded based on the immediate post-operative radiographs as anatomic (<1 mm of displacement), fair (1–3 mm of displacement), poor (>3 mm of displacement) (Matta and Tornetta III 1996). The Harris hip scale was used to assess the functional outcomes.

## Results

### Patient characteristics

Of 73 cases included in this study, 46 patients were operated through modified ilioinguinal approach combined with Kocher–Langenbeck approach (group A), whereas the other 27 patients were operated through the standard ilioinguinal approach combined with Kocher–Langenbeck approach (group B). No significant differences were observed between the groups in terms of gender, age and fracture types (p > 0.05) (Table 1).

**Table 1:** Patient demographics and characteristics

	Group A	Group B	p value
Number of case	46	27	
Gender: men/women	35/11	20/7	>0.05
Age in years (mean ± SD)	46.2 ± 3.12	44.9 ± 2.36	>0.05
Acetabular fracture type (Judet and Letournel)			
Transverse	13	8	>0.05
Associated transverse and posterior wall	7	4	>0.05
T-shaped	9	6	>0.05
Both column	17	9	>0.05

### Operative records

The mean operative time in group A was 123.2 ± 6.83 (102–207) min compared with 161.5 ± 8.16 (135–232) min in group B. The mean blood loss was 586.2 ± 56.3 (490–1350) ml in group A and 830.0 ± 82.0 (600–1500) ml in group B. The mean operative time and blood loss between the two groups were significantly different (p < 0.05) (Table 2).

**Table 2:** Surgical data of 73 patients with acetabular fractures involving two columns

	Group A	Group B	p value
Operation time (min)	123.2 ± 6.83	161.5 ± 8.16	<0.05
Blood loss (ml)	586.2 ± 56.3	830.0 ± 82.0	<0.05
Radiological end-result (residual displacement)			
Anatomic (0–l mm)	39 (84.8 %)	24 (88.9 %)	>0.05
Fair (l–3 mm)	5 (10.9 %)	2 (7.4 %)	
Poor (>3 mm)	2 (4.3 %)	1 (3.7 %)	
Functional result (Harris hip score)			
>80 points	37 (80.4 %)	22 (81.5 %)	>0.05
60–79 points	5 (10.9 %)	3 (11.1 %)	
<60 points	4 (8.7 %)	2 (7.4 %)	
Complication	10 (21.7 %)	9 (33.3 %)	<0.05
Superficial wound infection	0	1	
Lateral cutaneous nerve lesion	1	0	
Deep venous thrombosis	4	2	
Necrosis of the femoral head	2	2	
Heterotopic ossification	3	4	

### Radiographic assessment

In group A, 39 patients (84.8 %) had anatomic results, 5 patients (10.9 %) had fair results, and 2 patients (4.3 %) had a poor result. Meanwhile, anatomic results were achieved in 24 cases (88.9 %), fair results in 2 cases (7.4 %), and poor results in 1 case (3.7 %) in group B. The quality of reduction between the two groups was not significantly different (p > 0.05) (Table 2).

### Clinical outcome

In group A, Harris hip scale more than 80 points were obtained in 37 patients (80.4 %), 60–79 points in 5 patients (10.9 %) and less than 60 points in 4 patients (8.7 %). In group B, Harris hip scale more than 80 points were obtained in 22 patients (81.5 %), 60–79 points in 3 patients (11.1 %) and less than 60 points in 2 patients (7.4 %). The mean Harris hip scale scores between the 2 groups showed no significant difference between the groups (p > 0.05) (Table 2).

### Complications

The mean complication rate was 21.7 % (10 patients) in group A and 33.3 % (9 patients) in group B. In group A, osteonecrosis of the femoral head was observed in 2 cases. There were 4 deep vein thromboses documented with sonography. Heterotopic ossification was recorded in 3 patients. Lateral cutaneous nerve iatrogenic lesion resulted in 1 case and recovered after 6 weeks. In group B, Superficial infection was observed in 1 case. Osteonecrosis of the femoral head was observed in 2 cases. There were 2 deep vein thromboses documented with sonography. Heterotopic ossification was recorded in 4 patients. The mean complication rate between the 2 groups showed no significant difference between the groups (p > 0.05) (Table 2).

## Discussion

Open reduction internal fixation is gold standard and widely used for the treatment of displaced acetabulum fractures. Achieving good reduction of acetabular and satisfactory postoperative function is highly dependent on choosing the appropriate surgical approach. The operative approaches for treatment of displaced acetabular fractures includes: the anterior ilioinguinal approach, the posterior Kocher–Langenbeck approach, the extended iliofemoral approach and combined approaches. According to Judet and Letournel’s classification, there were five elementary fractures and five associated fractures. Based on this classification, Matta (1996) suggested a single surgical approach for six of ten acetabular fractures. Fracture of anterior wall, fracture of anterior column and fractures of the anterior column associated posterior hemi-transverse could be treated with the ilioinguinal approach. Fracture of posterior wall, fracture of posterior column, and fractures of the posterior column and posterior wall could be treated with the Kocher–Langenbeck approach. The surgical approach for the four remaining fracture types, transverse fracture, associated transverse and posterior wall fractures, T-shape fracture and both-column fractures was inconsistent. Those four type fractures involved two columns. Usually, the combination of an anterior and posterior approach should be considered when treat with those complex fractures.

In the 1960s, Letournel established the ilioinguinal approach for the treatment of pelvic ring and acetabular fractures (Letournel 1961). The ilioinguinal approach provides wide access to the anterior column of acetabulum. However, entire detachment of anterior part of the abdominal wall from the ilium or the inguinal ligament, may result in many soft tissue complications associated with this approach, such as postoperative wound infections, iatrogenic injury to the femoral nerve and the iliofemoral blood vessels (Matta 2006; Helfet et al. 1992). To reduce those complications, a modified ilioinguinal approach has been wide used currently (Yang et al. 2015). Using the modified ilioinguinal approach in combined surgical exposures may archive less complications and good functional outcomes in the management of acetabulum fractures involving two columns. However there is no clinical evidence to confirm its efficiency. The purpose of this study is to assess the operative time, blood loss, function outcomes and complications of modified ilioinguinal approach in combined surgical exposures for complex acetabular fractures management.

In this study, we firstly present our experience of using the modified ilioinguinal approach combined with K–L approach for acetabular fractures involving two columns. The main difficulty in this approach is to detach the iliopubic ligament. The middle iliopubic ligament is strong and difficult to dissect. A long sharp scissor can be used to release it from the iliopectineal eminence. After this stage, there is a subperiosteal “tunnel” between the two windows. The femoral vascular and iliopsoas muscles together with the femoral nerve as the whole bundle are retracted anteriorly and medially. The internal iliac fossa and lateral parts of the anterior column of acetabulum are exposured. In acetabular fractures, the primary objective is the perfect reduction of the both column. Reduction is facilitated with various techniques, including the use of clamps such as a modified Weber clamp, direct pressure with a Cobb elevator or ball-tipped spike pusher, traction with Schanz screws inserted into iliac crest or anterior inferior iliac spine percutaneously, the use of lag screws, reduction with a plate, and other reduction maneuvers that typically are used in acetabular surgery. Temporary K-wires fixations as a conventional and useful method allow maintaining the fracture reduction. In our opinion, this modified ilioinguinal approach offers satisfactory reduction and stable internal fixation for the anterior parts of the acetabular fracture patterns. More important, this approach avoids the surgical dissection of the soft tissue structures in the inguinal part and rectus abdominis. Therefore, this modified ilioinguinal approach is used for displaced acetabular fracture with the following advantages: (1) less invasive dissection without exposure of the femoral nerve, the external iliac vessels and lymphatic channels of the inguinal canal, which is associated with nerve injury, thrombosis and lymphedema, (2) less surgical duration, bleeding and postoperative complications such as heterotopic ossification, (3) preserving circulation of hip joint from ischemia, and (4) rectus abdominis intact offers additional benefits for early functional training. However, there is a risk to injury the corona mortis. For this reason, familiarity with the anatomic structure is essential before using this minimal invasive approach.

Due to the modified ilioinguinal approach mainly for the fractures of the anterior portion of acetabulum, the combined Kocher–Langenbeck approach should be considered for acetabulum fractures involving two columns. The indications for using the combined approach include most comminuted transverse fractures, associated transverse and posterior wall fractures, T-shape fractures and both-column fractures (Harris et al. 2008).

Our aim was to determine whether less complications and good functional outcomes of displaced acetabular fractures involving two columns, could be obtained by use of the modified ilioinguinal approach combined with K–L approach. In our study, 73 patients with acetabular fractures involving two columns were treated through combined approach by a single surgeon. Mean operative time was 123.2 min in group A as compared to 161.5 min in group B. Mean blood loss associated with approach was 586.2 ml in group A as compared to 830.0 ml in group B. The decreased operative time and blood loss are advantages of the modified ilioinguinal approach when combined with K–L approach for acetabular fractures involving two columns. An anatomical reduction was achieved in 84.8 % patients in group A which is comparable to the rates of 88.9 % in group B. The excellent functional outcome was achieved in 80.4 % patients in group A and in 81.5 % patients in group B. Those results were in keeping with previous studies (Andersen et al. 2010; Gary et al. 2012). The mean complication rate was 21.7 % in group A compared with 33.3 % in group B. The low incidence of surgical complications of the modified ilioinguinal approach combined with K–L approach was another important finding in our study. The major complication was deep venous thrombosis in both groups. Trauma was the main factor to deep venous thrombosis. Heterotypic ossification is another common complication, which is mainly due to posterior approaches. We emphasize that less invasive dissection and good reduction together with proper stabilization lead to early mobilization, less complications and good rehabilitation.

## Conclusions

This study provides evidence that modified ilioinguinal approach combined with Kocher–Langenbeck approach may be a good choice in the management of acetabular fractures involving two columns.

## References

[CR1] Andersen RC, O’Toole RV, Nascone JW, Sciadini MF, Frisch HM, Turen CW (2010). Modified stoppa approach for acetabular fractures with anterior and posterior column displacement: quantification of radiographic reduction and analysis of interobserver variability. J Orthop Trauma.

[CR2] Gary JL, VanHal M, Gibbons SD, Reinert CM, Starr AJ (2012). Functional outcomes in elderly patients with acetabular fractures treated with minimally invasive reduction and percutaneous fixation. J Orthop Trauma.

[CR3] Grubor P, Krupic F, Biscevic M, Grubor M (2015). Controversies in treatment of acetabular fracture. Med Arch.

[CR4] Harris AM, Althausen P, Kellam JF, Bosse MJ (2008). Simultaneous anterior and posterior approaches for complex acetabular fractures. J Orthop Trauma.

[CR5] Helfet DL, Schmeling GJ (1994). Management of complex acetabular fractures through single nonextensile exposures. Clin Orthop Relat Res.

[CR6] Helfet DL, Borrelli J, DiPasquale T, Sanders R (1992). Stabilization of acetabular fractures in elderly patients. J Bone Joint Surg Am.

[CR7] Jakob M, Droeser R, Zobrist R, Messmer P, Regazzoni P (2006). A less invasive anterior intrapelvic approach for the treatment of acetabular fractures and pelvic ring injuries. J Trauma.

[CR8] Kloen P, Siebenrock KA, Ganz R (2002). Modification of the ilioinguinal approach. J Orthop Trauma.

[CR9] Letournel E (1961). Fractures of the cotyloid cavity, study of a series of 75 cases. J Chronic Dis.

[CR10] Letournel E (1980). Acetabulum fractures: classification and management. Clin Orthop Relat Res.

[CR11] Letournel E (1993). The treatment of acetabular fractures through the ilioinguinal approach. Clin Orthop Relat Res.

[CR12] Matta JM (1994). Operative treatment of acetabular fractures through the ilioinguinal approach. A 10-year perspective. Clin Orthop Relat Res.

[CR13] Matta JM (1996). Fractures of the acetabulum: accuracy of reduction and clinical results in patients managed operatively within three weeks after the injury. J Bone Joint Surg Am.

[CR14] Matta JM (2006). Operative treatment of acetabular fractures through the ilioinguinal approach: a 10-year perspective. J Orthop Trauma.

[CR15] Matta JM, Tornetta P (1996). Internal fixation of unstable pelvic ring injuries. Clin Orthop Relat Res.

[CR16] Rommens PM, Broos PL, Vanderschot P (1997). Preparation and technique for surgical treatment of 225 acetabulum fractures. 2 year results of 175 cases. Unfallchirurg.

[CR17] Yang Y, Li Q, Cui H, Hao Z, Wang Y, Liu J (2015). Modified ilioinguinal approach to treat pelvic or acetabular fractures: a retrospective study. Medicine (Baltimore).

